# Research on stress performance of universally wrapped assembled joints of lattice wind power tower with concrete-filled steel tube

**DOI:** 10.1038/s41598-023-28929-x

**Published:** 2023-01-30

**Authors:** Yang Wen, Zhen Liu, Wen Xiong

**Affiliations:** School of Civil Engineering, Inner Mongolia University of Science and Technology, Baotou, 014010 Inner Mongolia China

**Keywords:** Engineering, Civil engineering

## Abstract

With the control parameters of the wall thickness of the ball table and the thickness of the pressure plate of the ball table, four universal wrapped assembled joint models were designed for the research of stress performance via static loading test. The failure mode of the joint, the axial force–displacement curve of the web member, the equivalent stress distribution of the ball table and the pressure plate of the ball table were analyzed, and the ABQAUS finite element software was used for the analysis of parameter expansion of the specimen. The research results show that the failure modes of universally wrapped assembled joints can be divided into the buckling failure of the ball table and the strength failure of the material. The wall thickness of the ball table is the key parameter affecting the failure mode of the joints, and the thickness of the pressure plate of the ball table is the crucial parameter influencing the ultimate bearing capacity of the joints. The maximum equivalent stress of the pressure plate and the ball table at compression zone for all the joints is occurred at the side squeezed by the bolt ball, and the growth rate of the equivalent stress for the ball table at compression zone is more uniform than that at tension zone. According to the finite element analysis results, when the wall thickness of the ball table is greater than 7 mm, the thickness of the pressure plate of the ball table is greater than 16 mm, the growth rate of the ultimate bearing capacity of the joint is significantly decreased, therefore, it is recommended to use the wall thickness of 7 mm and the pressure plate thickness of 16 mm in the actual design of the project.

## Introduction

In recent years, China's wind power industry has entered into the stage of high-speed development, which has become an extremely dynamic part of the global renewable energy construction. The installation scale of wind power in China has been leading in the world for many consecutive years^1,2^. With the increasing installed capacity of wind power, the requirements for wind power towers are getting higher and higher. The lattice tower with concrete-filled steel tube have been researched by the scholars from other countries.Liu Xiang and others^3^ analyzed the bearing capacity of the wind power tower with concrete-filled steel tube. The results show that all the performance indexes of the lattice tower with concrete-filled steel tube composite structure meet the force requirements; Wang Haijun and others^4^ carried out the wind-induced response analysis and mechanical performance analysis of the three-limb column lattice wind power tower with concrete-filled steel tube and the traditional conical single-tube tower. The results show that the lattice tower with concrete-filled steel tube is featured with the reasonable structural design, less steel consumption, better economy and good mechanical properties; Daniel and others^5^ studied the type of offshore wind power tower structure, and considered that the lattice tower structure are featured with the less steel consumption and one-time installation in place. Compared with the traditional cone wind power tower, lattice wind power tower will have a broad market with its advantages of convenient transportation, convenient installation, high stiffness and less steel^6–10^.

Being the connection part between the tower column and the web member of the lattice wind power tower as well as the important part of the structure of the lattice wind power tower, the joint is featured with the complex spatial coupling effect and complicated stress^11–14^. The intersecting joints and tube plate joints are mainly researched for the joint of lattice power tower currently. For example, Packer^15^ researched the ultimate bearing capacity of 31 joints with concrete filled circular steel tube at different forms, and obtained the calibration formula for the local compression bearing of the core concrete; Kim^16^ studied the static performance of the intersecting joints for rectangular steel tube, and proposed the best access range for the branch pipe, width-thickness ratio of chord rod section and the angle; Sakai^17^ carried out the monotonic static tests for three kinds of joints X, T and K with concrete filled square steel tube, proposed the calibration formula of the bearing capacity at different forms of joints and made the comparison; Qiao Ming^18^ carried out the experimental research and finite element analysis on the intersecting joints and tube plate joints of lattice wind power tower. The research indicated that the diameter ratio between the web member and the tower column and the diameter-thickness ratio of the tower column played a major role in the bearing capacity of the intersecting joint, and the thickness of joint plate could improve the bearing capacity of tube plate joint to a limited degree; Wen Yang and others^19^ conducted static load tests on four plate-insert joints with concrete filled steel tube, and completed the parameter expansion analysis with ABAQUS finite element software, the failure mode of the joints, the reasonable range of the thickness of the joint plate and the height of the spherical column were obtained. Li Bin and others^20^ carried out the parameter expansion research on the tube plate joints with ANSYS finite element software, and conducted the comparative analysis on the bearing performance of the tube plate joints with the changing parameters of the ratio of the diameter and thickness of the tower with concrete filled steel tube composite structure, the tower column limb diameter ratio of web member and concrete filled steel tube composite structure and the thickness of joint plate. The results showed that the radial stiffness and lateral loading resistance of the column limb with concrete filled steel tube could be improved from the composite structure filling the concrete into the steel tube. However, the above two types of nodes are disadvantageous at high-altitude welding, obvious stress concentration at the joints and difficult transportation, thus limiting the promotion and development of wind power industry.

On the basis, the bolt universal ball joints^21^, universal wrapped Y-joints^22^, and spherical plate branch joints^23^ have been proposed by the research group successively. The on-site welding, disassembly and assembly difficulties and other problems can be solved by the above joints, while the shear failure of the joint is caused for the intersection of the axis pulling and pressing the web member is outside of the tower column. Based on the previous research of the research group, a new type of universal wrapped assembled joint is designed creatively. The stress concentration issue at the connection of the joints can be solved for the axis pulling and pressing the web member of the joint lies in the axis of the tower column. With two control parameters of the thickness of pressure plate and the wall thickness of the ball table, the experimental study and finite element analysis of stress performance are carried out for four specimen models to obtain the influence law, joint stress distribution and failure mode of each factor.

## Testing survey

### Specimen design

Based on certain 1.5 MW wind power tower in Baiyun Obo mining area, the four-legged wind power tower with the latticed concrete filled steel tube was designed with SAP2000 software, and the internal force analysis was conducted. The calibration results can be shown in the Table 1, the internal force of the tower column is decreased constantly along with the increase of the number of tower layers, which is featured with the greater range and non-uniform distribution; The range of internal force value is small and the distribution of internal force is uniform relatively for the web member. Both of the internal force value and internal force ratio at the 18th layer are large, therefore, it is the weakest position for the tower at the 18th layer.

**Table 1:** Internal force value of bar.

Tier no.	Internal force value of bar						
	Cross section of tower column	Tension tower column	Compressed tower column	Cross section of web member	Tension rod	Compressed rod	Internal force ratio
1	φ400 × 20	4274	− 2694	φ140 × 10	140	− 151	1.08
2		4086	− 2617		170	− 205	1.20
3		3906	− 2489		172	− 200	1.16
4		3718	− 2402		176	− 202	1.15
5		3454	− 2202		175	− 205	1.17
6		3140	− 1914		174	− 207	1.19
7	φ350 × 15	2794	− 1877	φ140 × 10	175	− 207	1.18
8		2643	− 1654		180	− 204	1.13
9		2374	− 1530		175	− 205	1.17
10		2338	− 1367		172	− 185	1.07
11	φ300 × 10	2147	− 1311	φ140 × 10	177	− 180	1.02
12		1899	− 1190		174	− 182	1.05
13		1599	− 1146		175	− 182	1.04
14		1297	− 962		176	− 184	1.04
15		1034	− 906		178	− 186	1.04
16		894	− 761		180	− 191	1.06
17		454	− 560		195	− 184	0.94
18		312	− 321		182	− 219	1.20

The size verification is carried out at three parts of the tower (1–6 layers, 7–10 layers, 11–18 layers) for the cross-sectional dimensions of the tower column and the web member of the tower are different. The tower column and web member with the maximum internal force value at each part are selected for verification according to *Technical Specification for Concrete Filled Steel Tube Structure*^24^* and the Steel Structure Design Standard*^25^.

1. Tower calibration
  The tower column is concrete filled steel tube with large radial stiffness, the tensile and compressive bearing capacity shall be verified without the stability calibration. Seen from the Table 2, we can know that the tower columns at the 1st floor, 7th floor and 11th floor are calculated for the internal force values of the tower columns at these three floors are the maximum among each section of the tower, and the calibration results are shown in the table. The tensile and compressive bearing capacity are calculated according to Formulas (1) and (2) respectively:
  1 $$ N_{t} = f_{s} \cdot A_{s} $$
  2 $$ N_{u} = \phi_{1} \cdot \phi_{e} \cdot N_{0} $$

**Table 2:** Tower calibration.

Part	*N*_*t*_ /kN	*N*_*t*_*′*/kN	*N*_*u*_/kN	*N*_*u*_*′*/kN	Whether the design can be met
1	4274	5809	2694	11,932	Yes
2	2794	3901	1877	8828	Yes
3	2147	2297	1311	5821	Yes
  In the formula, $\phi_{{1}}$ is the slenderness ratio reduction coefficient; $\phi_{{\text{e}}}$ is the eccentricity reduction coefficient;$N_{0}$ is the axial compressive bearing capacity of short column,$N_{0} = 0.9 \cdot A_{c} \cdot f_{c} \cdot \left( {1 + \sqrt \theta + \theta } \right)$, wherein $A_{c}$ is the area of concrete section (mm^2^);$f_{c}$ is the compressive strength designed for concrete,$\theta$ is the confining coefficient of concrete,$\theta = A_{s} \cdot {f \mathord{\left/ {\vphantom {f {A_{c} \cdot f_{c} }}} \right. \kern-0pt} {A_{c} \cdot f_{c} }}$_._
2. Calibration of web member instability.
  The web member of wind power tower is hollow steel pipe, which is easy to lose stability, and its strength is decreased rapidly after the instability. Therefore, the stability of the web member is calculated. Seen from the Table 3, we can know that the web members of 6, 7 and 18 layers are selected for calibration. The calibration results are shown in the table.

**Table 3:** Calibration of web member instability.

Part	*N*/kN	Internal force value	Whether the design is unstable
1	360.00	207	No
2	372.37	207	No
3	396.49	219	No
  The following formula shall be satisfied for the stability bearing capacity of web member:
  3 $$ \frac{N}{\phi Af} \le 1.0 $$
  In the formula, *φ* is the stability coefficient.
  The results can meet the requirements via the calibration, and the design of tower size is reasonable.
  Specimen structure and field assembly drawing are shown in Fig. 1. Four joint models with the scale ratio of 1:1.6 are designed and made considering the internal force analysis, test site, test equipment capacity and others of SAP200 tower comprehensively, the size, structure and site assembly diagram of the specimen can be seen in Fig. 1. The wall thickness and pressure plate thickness of the ball table for JD-1–JD-3 are different, and JD-4 is the control test of JD-3. Each part of the specimen is prefabricated by the processing plant with consistent processing technology, and conveyed to the laboratory finally for the on-site assembly.

**Figure 1:**
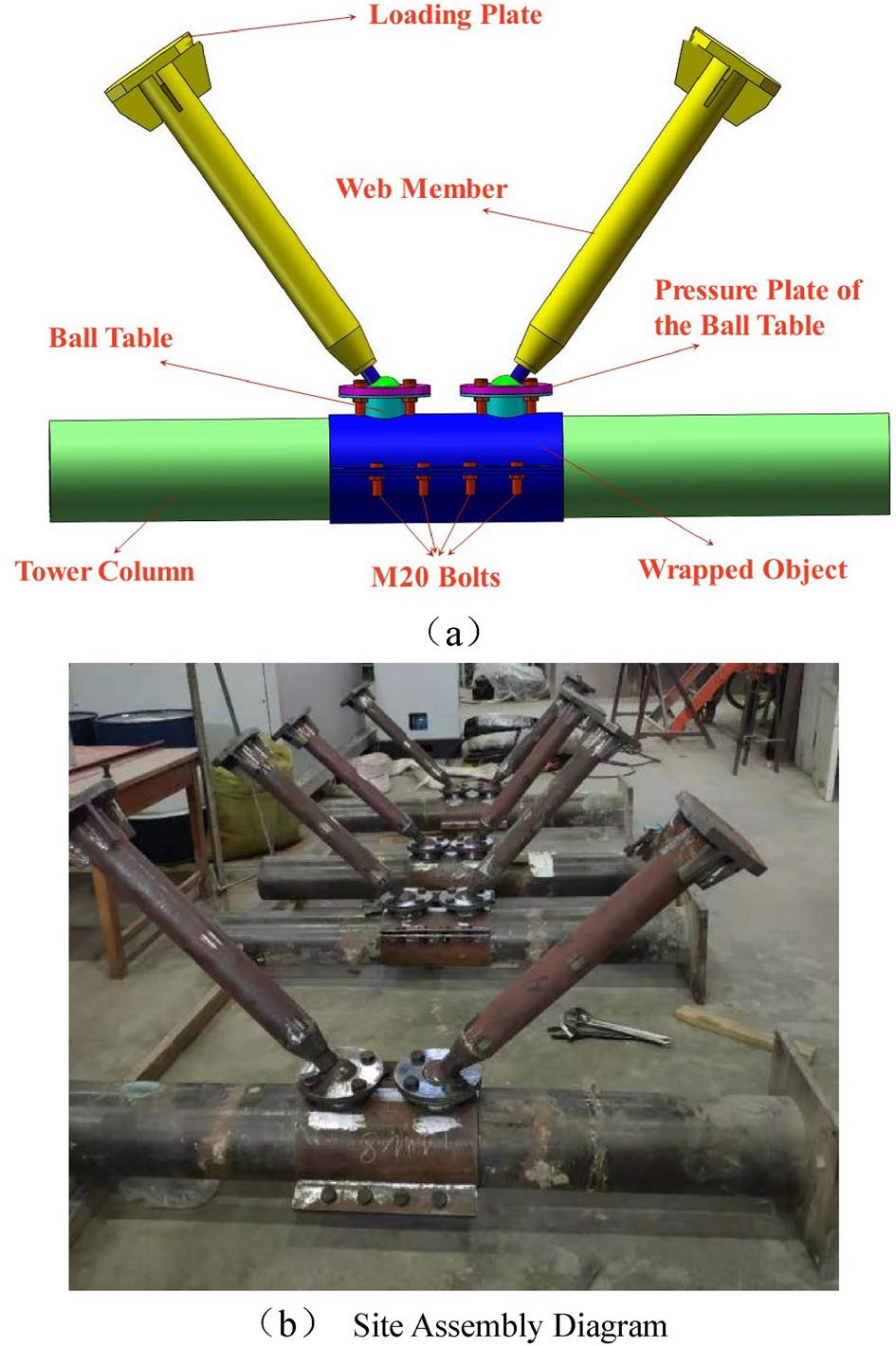
Specimen structure and site assembly drawing.
  The tower column is made of 20# seamless steel tube, and the internal is poured with the concrete at the strength grade of C40; the loading plate, web member, cone head, bolt ball, pressure plate of the ball table, ball table, wrapped object and steel tube of tower column are all made of Q235 steel; The two pieces of wrapped objects are processed and shaped by the steel plate bending technology at one time, the bolts of two wrapped objects, and between the plate of ball table and pressure plate are made of M20, and the bolts between the bolt ball and the web member are made of Grade-10.9 M30 high-strength bolts. When processing the material, the welds of loading plate, web member, cone head, ball table and wrapped object are groove welded according to *the Steel Structure Design Standard*^25^. Moreover, the effective thickness of welding is equal to that of the base metal. The geometric parameters of the components are shown in Table 4, and the mechanical property indexes of steel are shown in Table 5.

**Table 4:** Geometric parameters of components (*/*mm).

Joint number	Cross section of tower	Length of tower column	Cross section of wrapped object	Cross section of web member	Length of web member	Wall thickness of ball table	Thickness of pressure plate
JD-1	φ219 × 6	1800	φ235 × 8	φ89 × 5	700	8	10
JD-2	φ219 × 6	1800	φ235 × 8	φ89 × 5	700	8	16
JD-3	φ219 × 6	1800	φ235 × 8	φ89 × 5	700	4	16
JD-4	φ219 × 6	1800	φ235 × 8	φ89 × 5	700	4	16

**Table 5:** Mechanical property indexes of steel.

Parts	Thickness/mm	f_y_/MPa	f_u_/MPa	E_s_/MPa
Web member	5	348	482	2.02 × 10^5^
Pressure plate of ball table	10	332	485	2.05 × 10^5^
	16	336	473	2.04 × 10^5^
Ball table	4	342	462	1.98 × 10^5^
	8	347	466	2.01 × 10^5^
Wrapped object	8	318	466	1.99 × 10^5^
  The classified calculation is made for the web members and tower columns according to AISC 360-10^26^ as shown in Table 6. Based on the calculation, all the cross-sectional dimensions of the web members and the tower columns selected in the paper are compact.

**Table 6:** Slenderness limits.

Parts	Width-to-thickness	λp	λr
Web member	D/t	$0.11\frac{Es}{{fy}}$	
Tower	D/t	$0.07\frac{Es}{{fy}}$	$0.31\frac{Es}{{fy}}$

### Test device and loading system

**Figure 2:**
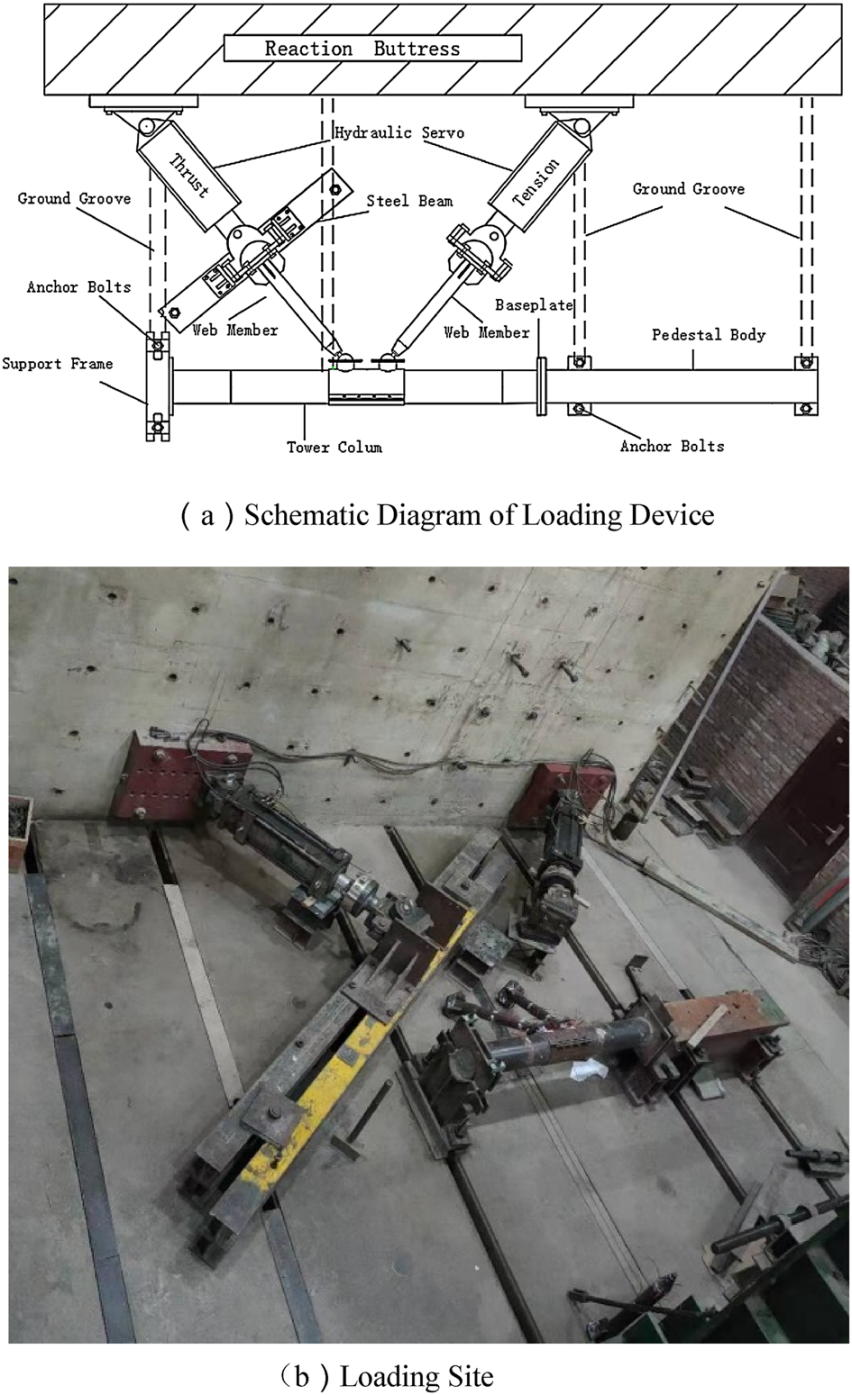
Loading device.

Considering the particularity of the joint model structure, the horizontal loading is adopted. One end of the tower column is fixed by the pedestal body, and the other is fixed by the support frame. The web member is connected with the hydraulic servo actuator through the loading plate. Two L-shaped steel plates are used on both sides of the compressive web member to provide lateral support. The steel plate is connected with the steel beam by bolts, the steel beam and the ground groove are fixed by anchor bolts to ensure that the load and the compression rod are always in a straight line and the eccentricity can be avoided. The loading device is shown in Fig. 2.

The theoretical ultimate bearing capacity of the joint model, the tension and compression borne by two web members respectively can be obtained from ABAQUS finite element analysis, and the theoretical ultimate bearing capacity is shown in Table 3. The compression and tension of two web members are loaded at the ratio of 1.2:1. The step-by-step loading is taken for the loading system. The pre-loading stage is conducted initially, which can be divided into three levels. 10% of the predicted ultimate bearing capacity is loaded at each level for 10 min, and it is unloaded at three levels after three-level loading is completed; In the standard loading stage, the load of each level is also 10% of the predicted ultimate bearing capacity step by step. When 75% of the predicted ultimate bearing capacity is reached or there is obvious plastic deformation at the joint, 5% of the predicted ultimate bearing capacity is taken for the loading at each level in order to get the more accurate results until the specimen is loaded to the failure.

## Measuring content and measuring point arrangement

**Figure 3:**
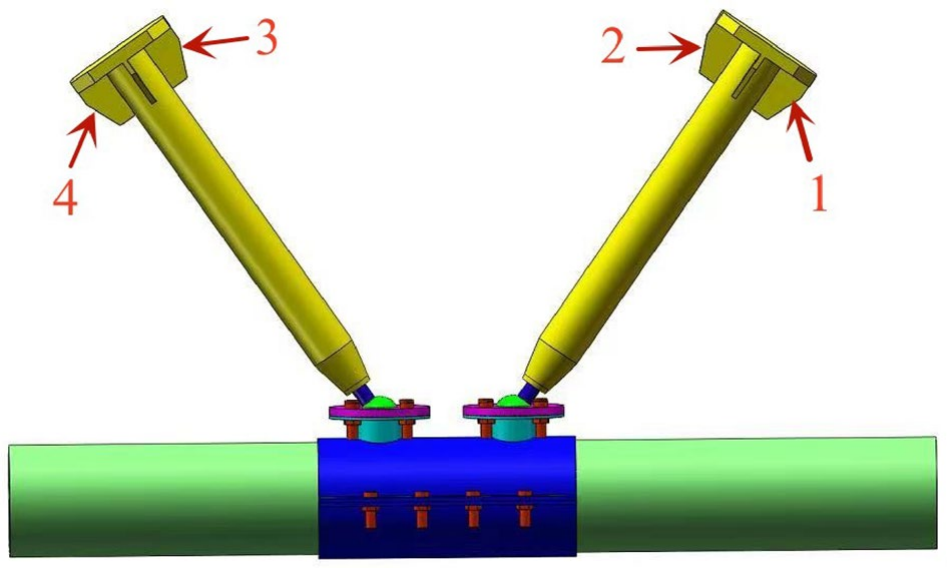
Layout of displacement gauge.

**Figure 4:**
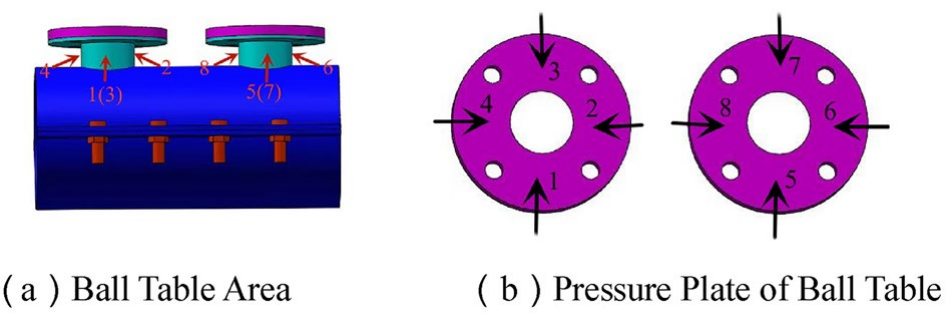
Layout of strain gauge.

No.1-4 displacement gauges are set at the end to pull and press the web member in order to obtain the axial force–displacement curve of the web member. A set of rosette gauges are arranged at the pressure plate and the area of the ball table at every 90° to count the strain distribution and growth of the pressure plate and ball table; The layout of displacement gauge and strain gauge is shown in Figs. 3 and 4.

### Test phenomenon and failure mode

#### Test phenomenon

There is no obvious test phenomenon at the beginning of loading for JD-1–JD-4. When the tension for specimen JD-1 is added to 154 kN, the out-of-plane deformation of the pressure plate at tension zone increases gradually, and the bolt tends to be pulled out. When the final tension is added to 180 kN, the pressure plate of the ball table at tension zone is pulled into an ellipse, and the bolt ball at tension zone is pulled out, and the test is ended. The case for specimen JD-2 is similar with that of specimen JD-1. When the final tension is increased to 319 kN, the pressure plate mouth of the ball table at tension zone is pulled into an obvious ellipse, and the bolt ball at tension zone is pulled out, and the test is ended. When the tension for specimen JD-3 is increased to 214 kN, there is a clear gap between the table plate and the pressure plate of the ball table at tension zone, and the small deformation is occurred at the axial direction of the tower column at the ball table at compression zone; When the tension is increased to 242 kN finally, it is broken suddenly at the crown point of the ball table at tension zone, and the test is ended. The case for specimen JD-4 is similar to that of specimen JD-3. When the tension is increased to 229 kN finally, it is broken suddenly at the crown point of the ball table at tension zone, and the test is ended.

#### Failure mode

**Figure 5:**
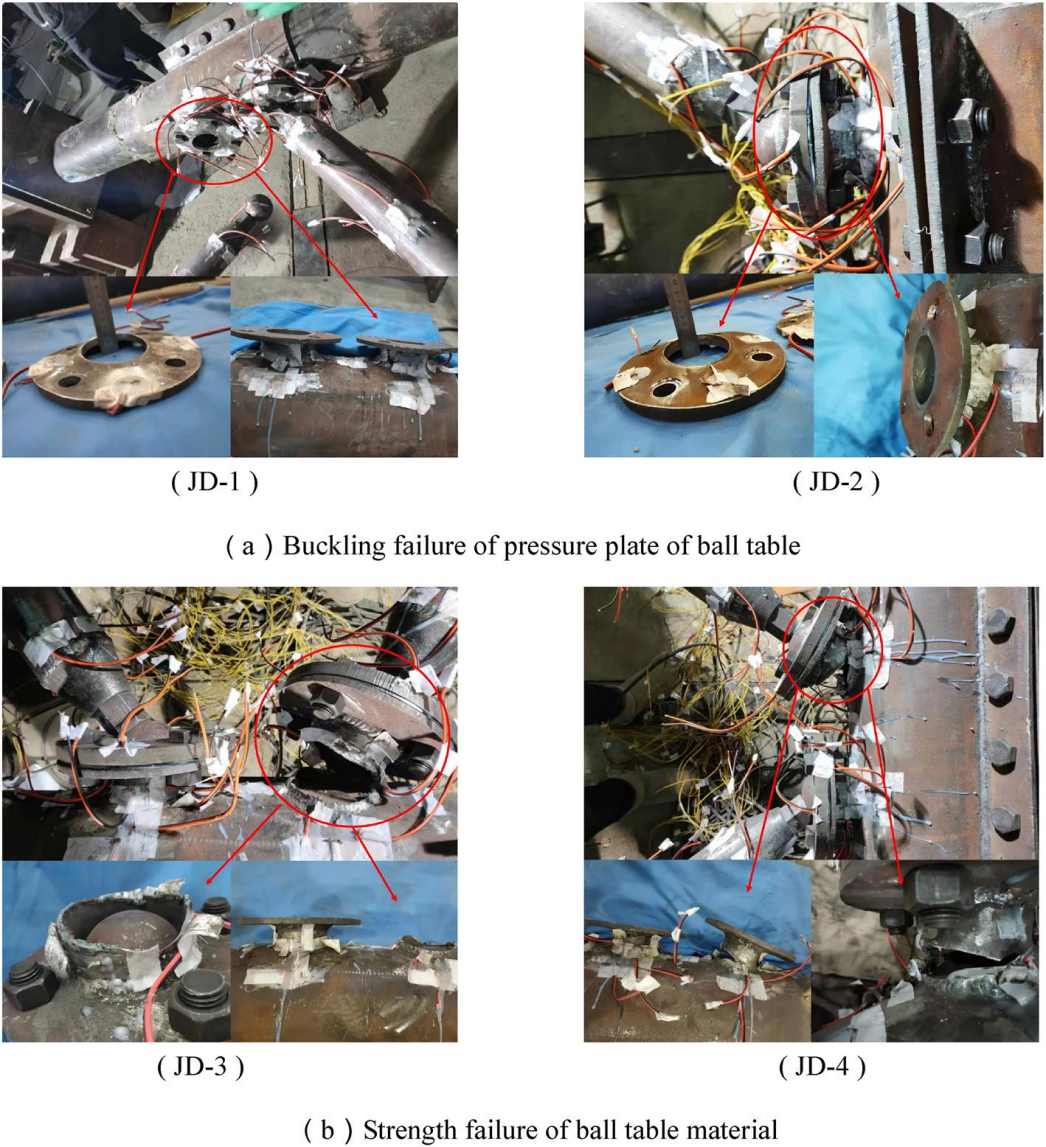
Failure mode.

There are two failure modes for the above four joints (Fig. 5). Specimen JD-1 and JD-2 are the buckling failure mode of the pressure plate of the ball table, specimen JD-3 and JD-4 are the strength failure mode of the ball table material.

When the wall thickness of the ball table for specimen JD-1 and JD-2 is 8 mm, the damage and large deformation do not occur in the ball table, and the ultimate bearing capacity of the specimen depends on the binding force of the pressure plate of the ball table on the bolt ball at tension zone. Along with the increase of loading, the pressure plate of the ball table at tension zone gradually buckles along the axial direction of the ball table. The pressure plate mouth of the ball table at tension zone is gradually pulled into an ellipse, thus reducing the binding force of the pressure plate of the ball table at tension zone on the bolt ball and finally pulling out the bolt ball. In this process, the deformation of the pressure plate of the ball table develops slowly, therefore, the buckling failure mode of the ball table is a kind of plastic failure. For specimen JD-3 and JD-4, the wall thickness of the ball table is 4 mm and the pressure plate of the ball table is 16 mm. Compared with the thinner wall thickness of the ball table, the thickness of the pressure plate of the ball table is enough to restrict the bolt ball to be pulled out. With the gradual increase of loading, there is no obvious deformation trend for the pressure plate of the ball table. Finally, when the ultimate strength is reached in the ball table, the ball table is pulled off, and the test is ended. It is 35° between the stress direction of the ball table and the axial direction of the ball table, where the ball table is broken quickly with a flat fracture by the greater shearing stress. The failure phenomenon is extremely sudden without obvious failure precursor, therefore, it is a kind of brittle failure for the strength failure mode of the ball table material.

Seen from the test results, we can know that there is no significant difference in the failure mode of the specimen JD-1 and JD-2 when the thickness of ball table wall is unchanged, the ultimate bearing capacity of ball table and the radial stiffness of the ball table are large, and the thickness of the compressive plate of ball table is increased from 10 to 16 mm; The failure mode of specimen JD-2, JD-3 and JD-4 is changed from the failure mode of material strength of ball table to the buckling failure mode of compressive plate of the ball table, and the specimen is transformed from the brittle failure to the plastic failure when the thickness of the compressive plate of the ball table is unchanged and has enough binding force on the bolt ball, and the thickness of the ball table wall is increased from 4 to 8 mm.

## Test results and analysis

### Axial force–displacement curve of assembled joint web member

**Figure 6:**
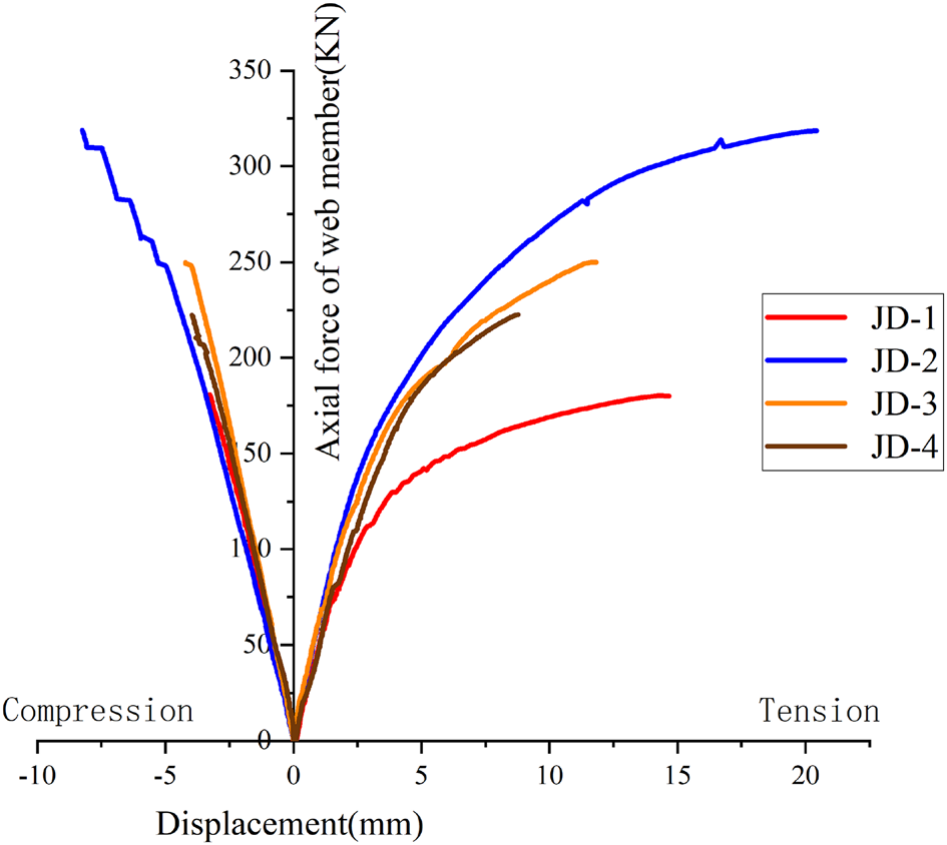
Axial force–displacement curve of web member.

**Figure 7:**
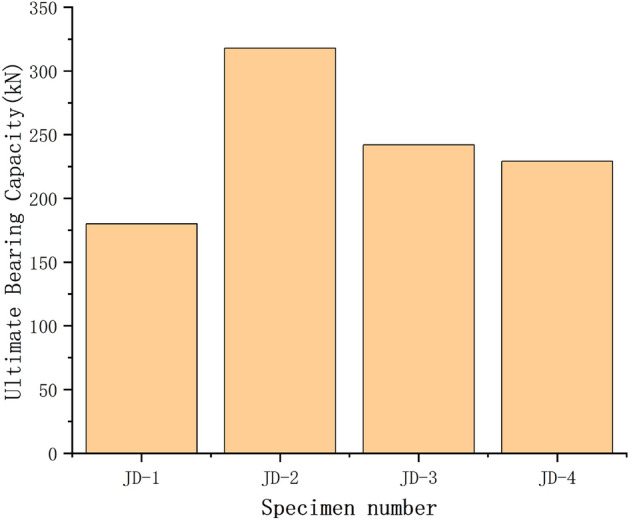
Ultimate bearing capacity of specimen.

It can be seen from Fig. 6 that the curves of web member at the tension zone and compression zone of JD-1–JD-4 are featured with linear increase at the initial stage of loading, and the curves are consistent basically; With the increase of loading, the curve of web member at compression zone is still a straight line, and the slopes of different curves are still similar; while the curve of the web member at tension zone appears inflection point in succession, and the slope is different at various degrees. There are inflection points in the curve of JD-1 and JD-2 when the tension is increased to 105 kN and 126 kN respectively; the slope of the curve decreases significantly when the tension reaches 134 kN and 254 kN respectively; Finally, the slope of the curve approaches zero when the tension reaches 180 kN and 318 kN respectively, and the test is ended. The slope of JD-3 decreases gradually along with the increase of the tension, and the test is ended until the tension is increased to 242 kN. The case of JD-4 is similar to that of JD-3, and the test is ended when the load is increased to 229 kN. The ultimate bearing capacity of the specimen can be seen in Fig. 7.

Concerning the web member curves at the compression zone of specimens JD-1–JD-4, the deformation when compressing and tensing the web member of the specimen is not coordinated. It is because that the radial stiffness of the tower column filled with the concrete is improved greatly, the ball table at the compression zone only bears a small part of the pressure, and most of the pressures are transmitted to the tower column from the compressed web member, high-strength bolt and bolt ball for dissipation, the compression zone is not damaged, and the ultimate is not reached.

Via the comparison among JD-1–JD-4, the wall thickness of the ball table is 8 mm, the slope of axial force–displacement curve for the web member at tension zone increases when the thickness of the pressure plate of the ball table is increased from 10 to 16 mm, thus indicating that the increase of the pressure plate of the ball table can improve the ultimate bearing capacity of the specimen greatly, while the deformation capacity of the joint can not be enhanced; When the thickness of pressure plate of the ball table is 16 mm, and the wall thickness of the ball table is increased from 4 to 8 mm, the elastic–plastic stage of the curve increases. The increase of the wall thickness of the ball table can effectively improve the overall deformation capacity of the specimen structure.

### Initial stiffness and ductility factor

**Figure 8:**
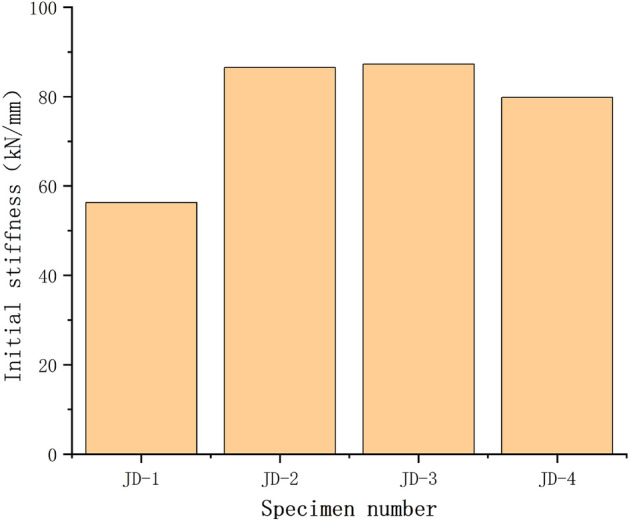
Initial stiffness.

The initial stiffness of the joint obtained by the secant slope corresponding to the elastic limit of each joint can be shown in Fig. 8. For the specimens JD-1–JD-4, when the thickness of the ball table wall is 8 mm, the ball table pressure plate is increased from 10 to 16 mm, the initial stiffness of the specimen is improved; when the bearing plate is 16 mm and the thickness of the bearing wall is increased from 4 to 8 mm, the initial stiffness of the specimen has no significant difference.

**Figure 9:**
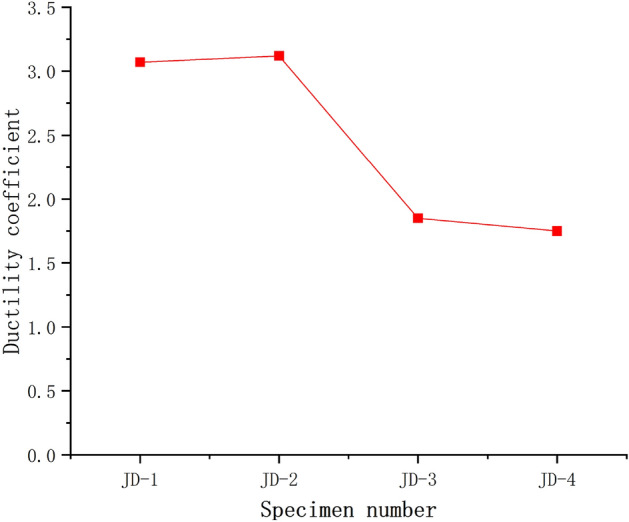
Ductility factor of specimen.

Figure 9 shows the ductility factor of specimens calculated by geometric method, it can be seen from the comparison of specimen JD-1 and JD-2 that the change of the ductility factor is not significant when the thickness of the ball table wall is 8 mm and the compressive plate of the ball table is increased from 10 to 16 mm; the ductility factor of the specimen is increased by 68.6% in the comparison of specimen JD-2 and JD-3 when the compressive plate of the ball table is 16 mm and the thickness of ball table wall is increased from 4 to 8 mm. It can be seen from the comparison of specimen JD-1–JD-4 that the increase of the thickness of compressive plate of the ball table has little influence on the ductility factor of the specimen; and the ductility of the specimen can be better played via the increase of the thickness of ball table wall. The thinner wall thickness of the ball table breaks the node ball table suddenly and quickly, which also reflects that the wall thickness of the ball table is the key parameter affecting the change of the failure mode of the node.

### Equivalent stress curve of ball table

The ball table plays a vital role for the joint to fix the bolt ball. The ball tables at compression zone and tension zone are severely squeezed by the bolt ball with extremely complex stress mode. The equivalent stress curve of the ball table zone (see Fig. 4a for strain measuring points) is shown in Fig. 11.

**Figure 10:**
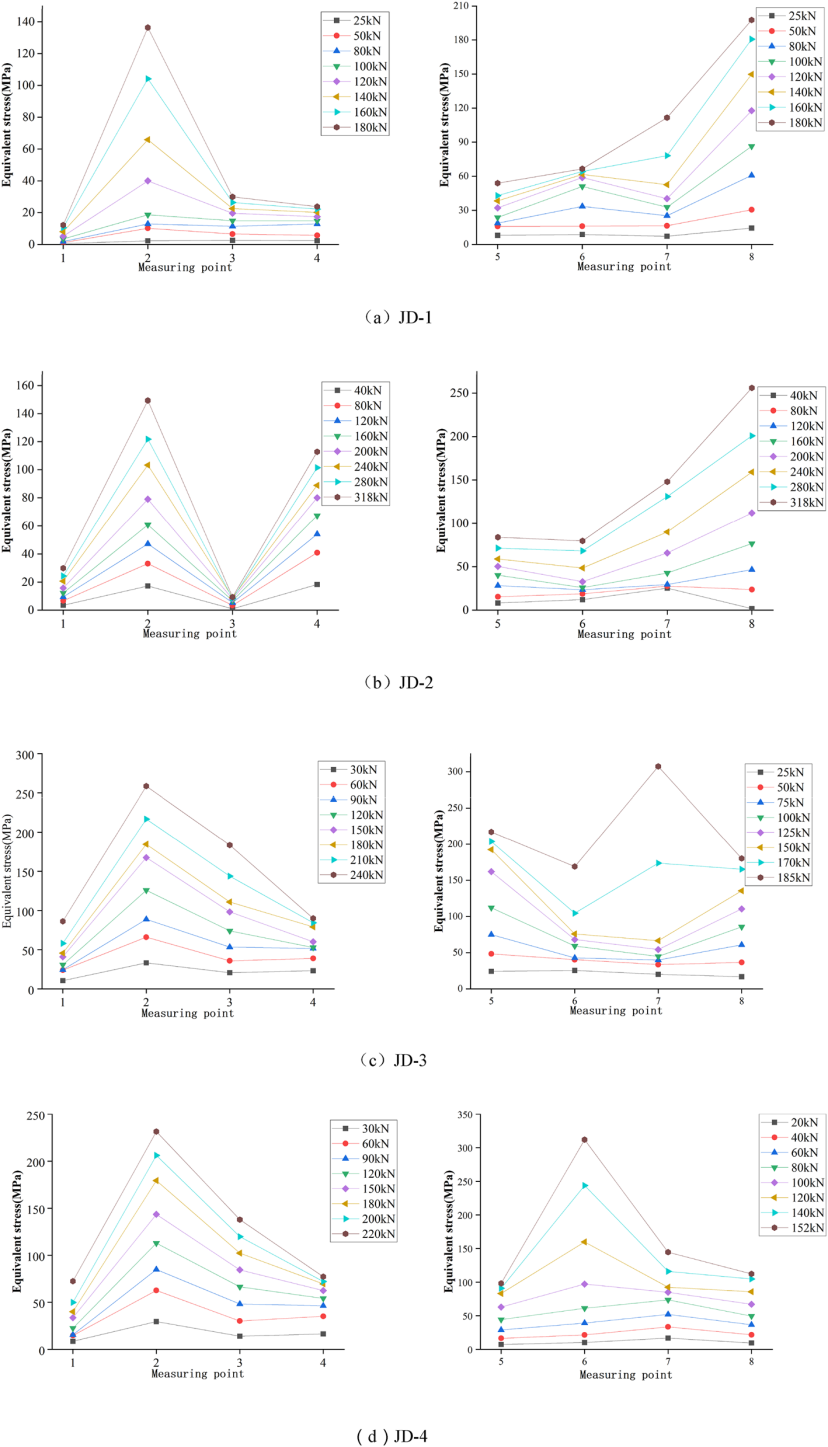
Equivalent stress curve of the ball table at compression zone and tension zone.

As shown in Fig. 10, the maximum equivalent stress of the ball table at compression zone is occurred at No.2 measuring point for JD-1–JD-4, and the values are 139 MPa, 154 MPa, 259 MPa and 243 MPa respectively; the maximum equivalent stress of the ball table at tension zone is occurred at No.8 measuring point for JD-1 and JD-2 with the value of 201 MPa and 252 MPa respectively, which is at No.7 and No.6 measuring points for JD-3 and JD-4 respectively with the value of 311 MPa and 313 MPa respectively. The minimum equivalent stress of the ball table at compression zone is occurred at No.1 measuring point for JD-1, JD-3 and JD-4 with the value of 17 MPa, 81 MPa and 73 MPa respectively, and that for JD-2 is occurred at No.3 measuring point with the value of 17 MPa; the minimum equivalent stress of the ball table at tension zone is occurred at No.5 measuring point for JD-1 and JD-4 with the value of 56 MPa and 99 MPa respectively, that for JD-2 and JD-3 is occurred at No.6 measuring point with the value of 85 MPa and 168 MPa respectively. The range (the difference between the absolute value of the maximum and minimum equivalent stress) of equivalent stress for JD-1–JD-4 is 122 MPa, 145 MPa, 137 MPa,167 MPa,78 MPa,143 MPa,170 MPa and 214 MPa respectively. At the beginning of loading, the equivalent stress distribution and stress growth range of JD-1–JD-4 at compression zone and tension zone of the ball table are relatively uniform. The growth of equivalent stress at No.2 and No.8 measuring points is rapid along with the gradual increase of the compression and tension for JD-1, which is caused by the occurred stress concentration phenomenon when the loading is increased; A slight leap of stress is occurred at No.2 measuring point when the stress is increased to 100 kN; With the continuous increase of loading, the equivalent stress increases uniformly until the end of specimen loading; the equivalent stress distribution and stress growth range of the ball table at compression zone and tension zone for JD-2 in the whole loading process are relatively uniform; the growth range of the equivalent stress at No.2 and No.3 measuring points on the ball table at compression zone for JD-3 and JD-4 is greater along with the increase of loading. The leap phenomenon of stress is occurred at the corresponding No.6 and No.7 measuring points when the tension is increased to 150 kN and 100 kN respectively, for the stress of the ball table at tension zone is rearranged caused by the deformation of the table plate at tension zone. The equivalent stress continues to increase along with the loading increase until the yield strength is reached in the ball table.

Via the comparison among JD-1–JD-4, the maximum equivalent stress of the ball table at compression zone is occurred at No.2 measuring point where the side is squeezed by the bolt ball, the maximum equivalent stress of the ball table at tension zone is occurred at No.8, No.8, No.7 and No.6 measuring point respectively. The growth range of the equivalent stress curve for the ball table at compression zone is more uniform than that at tension zone. In conclusion, the thickness of the ball table wall is the key parameter affecting the distribution of the equivalent stress and the growth trend of the equivalent stress in the compression and tension zone. The maximum equivalent stress of the ball table in the compression zone and the tension zone is decreased along with the increase of the thickness of ball table wall. The majority of the load transmitted by the bolt ball in the compression zone is dissipated by the tower column, and the stress concentration of the ball table in the compression zone is not obvious. The load of the ball table in the tension zone is mostly borne by the ball table itself. The stress concentration of the ball table in the tension zone is very obvious due to the large loading, therefore, the equivalent stress range of the ball table in the compression zone is decreased with the increase of the thickness of the ball table wall, while that in the tension zone is increased along with the increase of the thickness of the ball table wall.The increase of the thickness of the compressive plate of the ball table greatly increases the ultimate bearing capacity of the joint. The maximum value of the equivalent stress and the range of the equivalent stress of the ball table at the compression zone and tension zone are improved slightly caused by the larger loading. The fluctuation of the equivalent stress of the ball table at different degrees in the tension zone is caused by the deformation of the ball table plate in the tension zone. It is suggested to thicken the ball table plate in the tension zone at the practical engineering.

### Equivalent stress curve of pressure plate of the ball table

**Figure 11:**
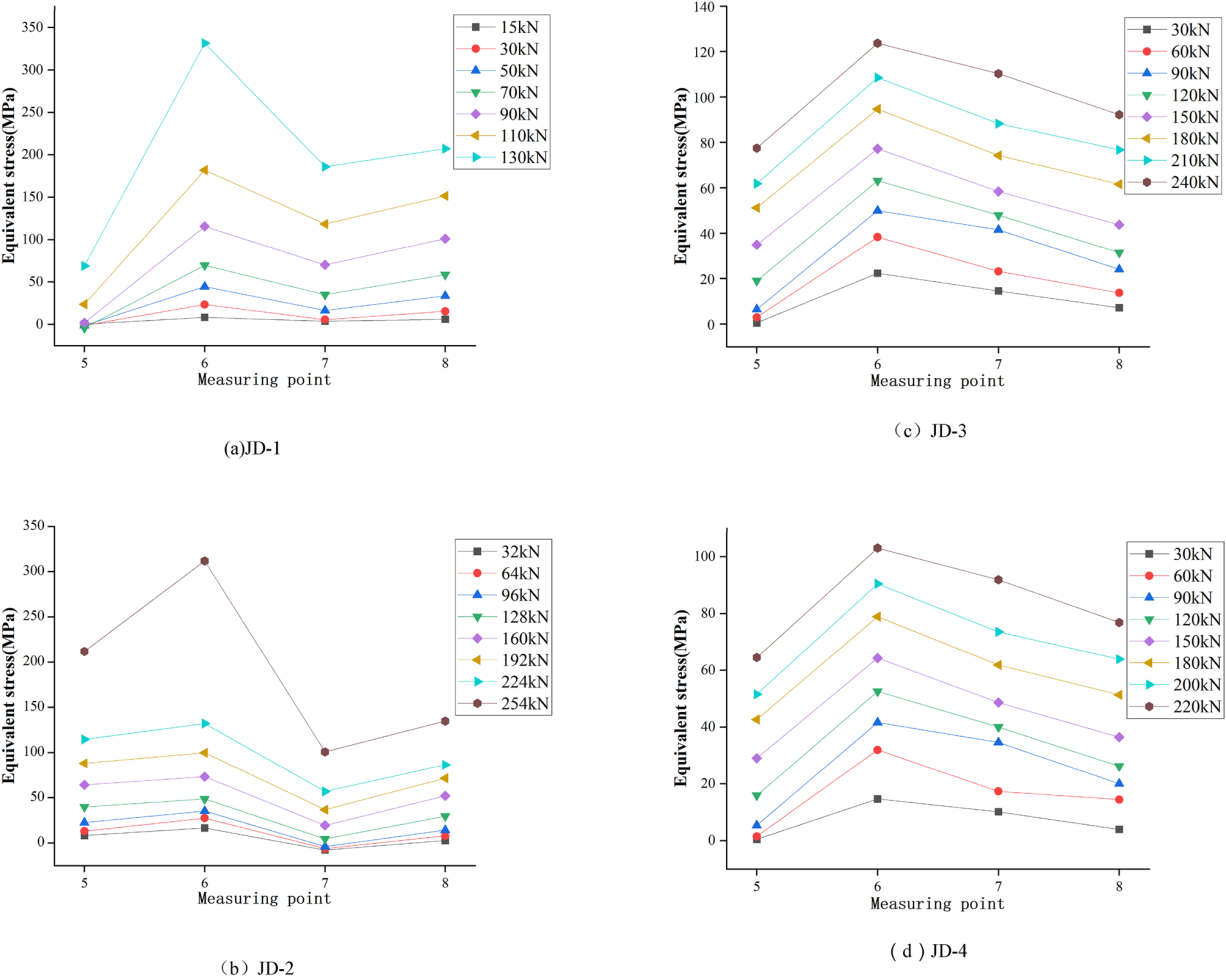
Equivalent stress curve of pressure plate of the ball table at tension zone.

There is less stress for the pressure plate in the ball table at compression zone, which mainly plays a structural role, therefore, the detailed comparison is not displayed in the paper. The equivalent stress curve of the pressure plate (see Fig. 4b for strain measuring point) in the ball table at tension zone can be seen in Fig. 11.

As shown in Fig. 11, the equivalent stress at No.6 measuring point is the maximum of the pressure plate of the ball table at tension zone for JD-1–JD-4 with the value of 341 MPa, 329 MPa, 122 MPa and 107 MPa respectively. The minimum equivalent stress for JD-1, JD-3 and JD-4 is occurred at No.5 measuring point with the value of 71 MPa, 79 MPa and 62 MPa respectively. The minimum equivalent stress for JD-2 is occurred at No.7 measuring point with its value of 117 MPa; the equivalent stress range for JD-1–JD-4 is 270 MPa, 212 MPa, 43 MPa and 45 MPa respectively. At the beginning of loading, the equivalent stress curve at tension zone grows more evenly. A slight leap of stress is occurred at No.6, No.7 and No.8 measuring points when the tension is increased to 70 kN for JD-1. The deformation of the table plate and the growth range of the equivalent stress for the pressure plate for the ball table at tension zone are increased gradually along with the increase of loading until the pressure plate yields. The case for JD-2 is similar to that of JD-1. A great leap is occurred at No.5 and No.6 measuring points, a slight leap is occurred at No.7 and No.8 measuring points when the tension is increased to 224 kN until the pressure plate yields. The equivalent stress curve for the pressure plate of the ball table at tension zone for JD-3 and JD-4 grows steadily without significant leap phenomenon in the loading process.

Via the comparison among JD-1–JD-4, the wall thickness of the ball table is 16 mm, the maximum equivalent stress of the pressure plate of the ball table at tension zone is improved by 1.7 times and the equivalent stress range is increased by 3.9 times when the wall thickness of the ball table is added from 4 to 8 mm. When the wall thickness of the ball table is 8 mm, the maximum equivalent stress of the pressure plate of the ball table at tension zone is decreased by 3.5% and the equivalent stress range is decreased by 21.5% when the thickness of the pressure plate of the ball table is added from 10 to 16 mm. The maximum equivalent stress for four joints is occurred at No.6 measuring point where the side of the pressure plate of the ball table at tension zone is squeezed by the bolt ball. In conclusion, the thickness of the compressive plate and the thickness of the ball table wall are the key parameters affecting the equivalent stress distribution and equivalent stress growth of the compressive plate of ball table in the tension zone. The ultimate bearing capacity of the joint is improved slightly caused by the increase of the thickness of ball table wall, and the larger loading improves the maximum equivalent stress of the compressive plate of ball table in the tension zone greatly. Meanwhile, the equivalent stress range of the compressive plate of ball table at the tension zone is increased significantly due to the stress concentration; the ultimate bearing capacity of the joint is improved significantly by the increase of the thickness of compressive plate of the ball table, the overall equivalent stress level is reduced slightly, and the stress concentration phenomenon is significantly weakened. The increase of the thickness of the ball table wall can improve the material utilization rate of the compressive plate of ball table, while it can aggravate the stress concentration phenomenon. The increase of the thickness of the compressive plate of ball table can make the equivalent stress distribution of the compressive plate of ball table in the tension zone more uniform, and make the material utilization more sufficient. Therefore, it is suggested to increase the thickness of compressive plate of the ball table as much as possible under the premise that the bearing capacity can be met by the ball table. The equivalent stress level of No.6 measuring point is higher with faster growth of equivalent stress, and it is easy for the side of compressive plate of ball table at the tension zone squeezed by the bolt ball to have the out-of-plane deformation caused by the greater stress, and it is recommended to set up the reinforcement device at the side of the compressive plate of ball table at the tension zone squeezed by the bolt ball in the practical engineering.

### Finite element analysis

On the basis of the previous scale model test of the joint, the three-dimensional modeling of the joint is carried out with SolidWorks structural design software. The property of the related materials for the model is defined with ABAQUS software. The appropriate contact and boundary conditions of the parts are set with the reference of the combination of the components. The unit property is selected reasonably for meshing according to the features of different parts, the load conditions and analysis steps are accurately defined, and the nonlinear finite element analysis is carried out in order to obtain more realistic, reasonable and reliable modeling results.

The setting of related parameters of the model is calculated with ABAQUS software via the comparison of the failure mode and equivalent stress distribution of the joints obtained from the finite element simulation and field modeling test, the key parameters of the joint are selected for the expansion analysis to make up for the defect that the more accurate conclusions can not be obtained due to the less samples of the field test, and obtain the optimal value interval of key parameters, thus laying the foundation for the in-depth research of such joint later.

### SolidWorks modeling

**Figure 12:**
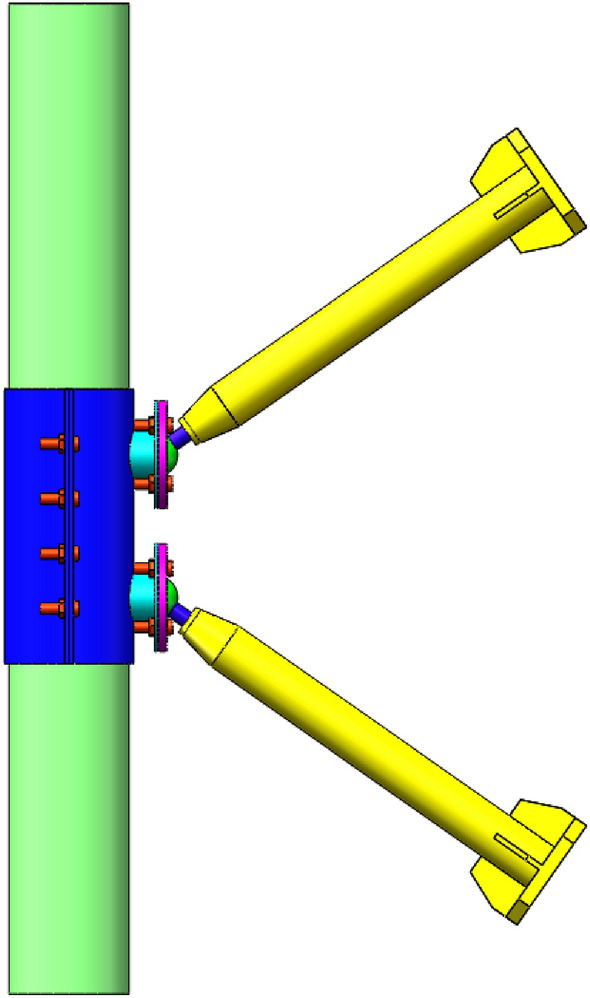
Joint assembly diagram (SolidWorks 2020 https://www.3ds.com/products-services/simulia/).

The SolidWorks assembly diagram is shown in Fig. 12. Each part is modeled according to the actual specimen size of 1:1. The format is conversed and imported into ABAQUS for further deepening after the assembly is completed, and the nonlinear finite element analysis is completed.

Compared with the built-in modeling module in ABAQUS, SolidWorks software can provide a more simple and convenient solid modeling function, which can carry out the design work of stretching, rotating, thin-walled, cutting and drilling based on the characteristics of parts accurately, and assemble various parts intelligently via capturing the relationship among the objects, convert from two-dimensional graphic design to three-dimensional solid design rapidly, realize the change of parameter size quickly in the later research process of parameter expansion, and improve the efficiency of simulation work.

### ABAQUS model deepening

#### Material constitutive

All of the steel parts of tower column, wrapped object, ball table and compressive plate of ball table in the paper are carbon steels with significant yield platform, therefore, the secondary yielding flow model given by Han Linhai^26^ is used for simulation, the concrete stress–strain relationship model proposed by Han Linhai^27^ is used for the core coagulation in column, and the plastic damage model^28^ is used for definition at the plastic stage of concrete.

#### Contact and boundary conditions

The joint tower column interacts with the core concrete in the form of "hard touching" and "penalty friction", and the friction coefficient is taken as 0.4. The bolt ball interacts with the ball table, pressure plate of the ball table, and the upper wrapped object in the form of "hard touching" and "penalty friction", and the friction coefficient is taken as 0.15. Other contacts are defined by Tie binding.

**Figure 13:**
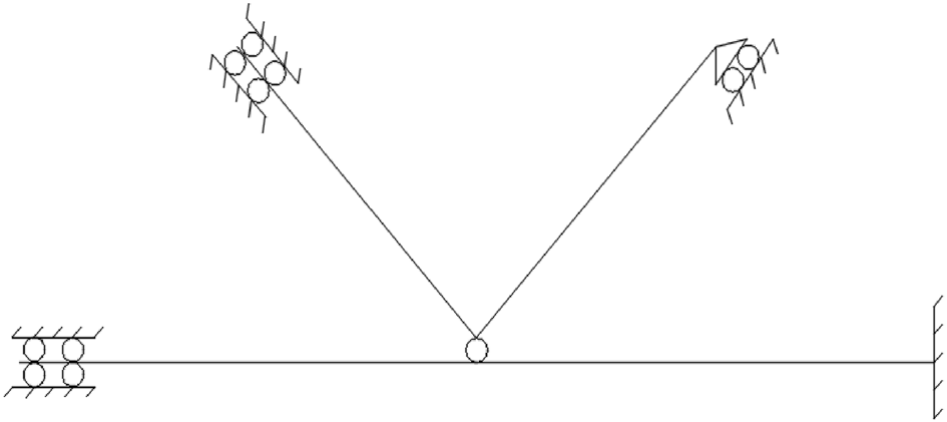
Boundary conditions.

The boundary conditions of the model are set according to the test. The fixed and directional hinge are taken for the simulation of two sides of the tower respectively. The hinge is taken for the loading side of the web member at the tension zone, and the directional hinge is used in the loading side of the compression rod. The boundary conditions are shown in the Fig. 13.

### Unit selection and mesh subdivision

**Figure 14:**
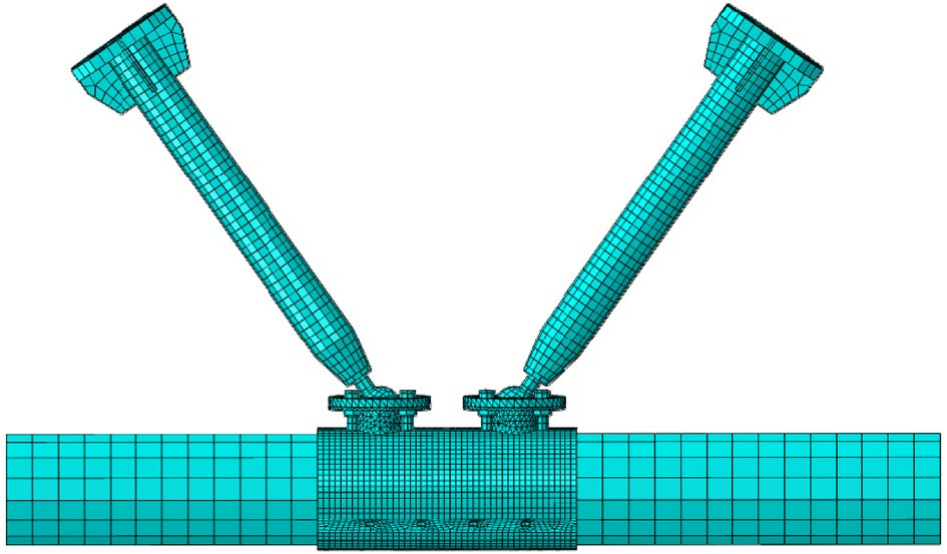
Mesh subdivision (ABAQUS 2020 https://www.3ds.com/products-services/simulia/).

The unit type is set with C3D8R for the core concrete, C3D8I is used for conventional steel parts, and C3D10 is used for the ball table, ball table plate and compressive plate of ball table. The mesh subdivision is shown in Fig. 14.

### Node finite element analysis

#### Calibration of failure pattern

**Figure 15:**
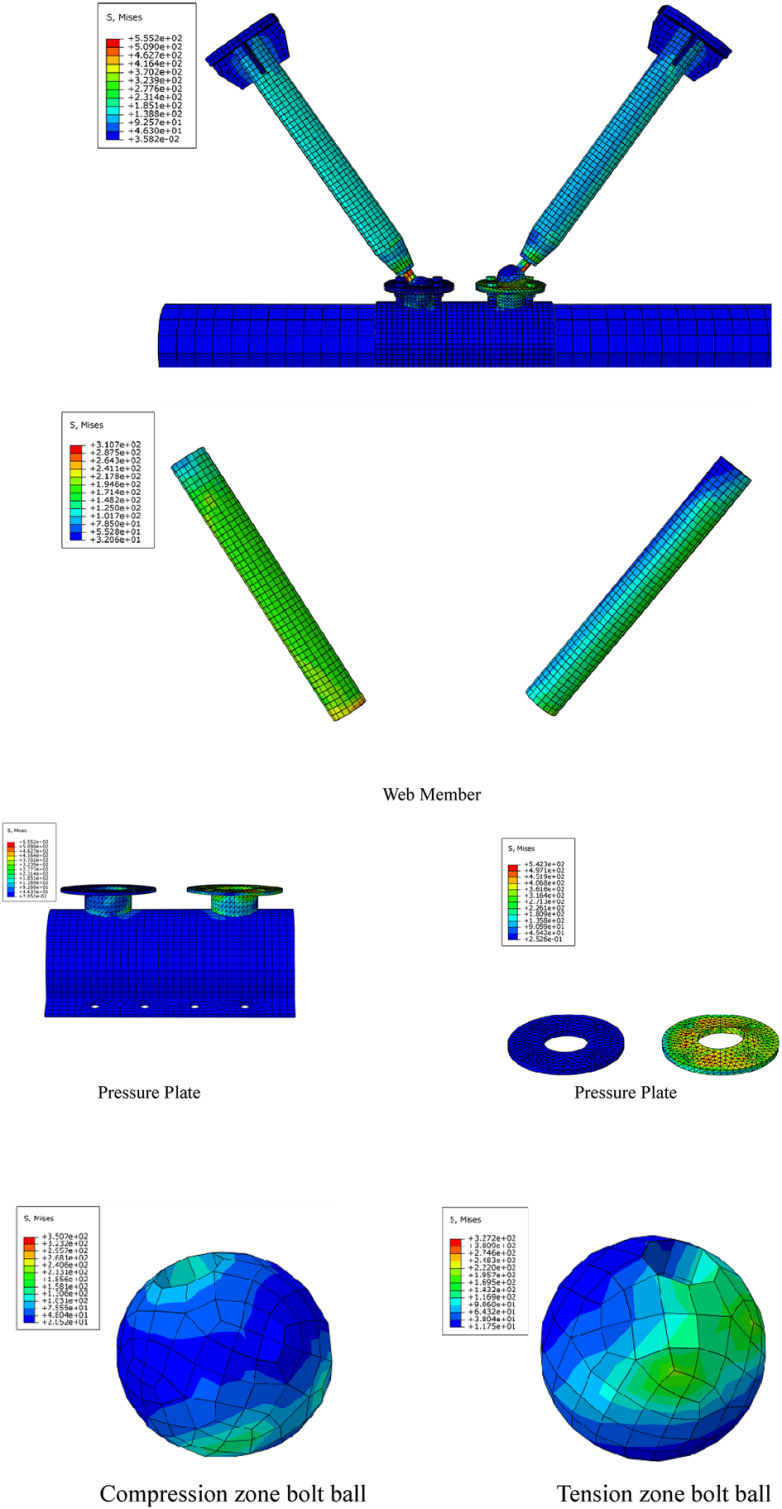
Stress cloud chart (ABAQUS 2020https://www.3ds.com/products-services/simulia/).

With the example of the stress cloud chart of specimen JD-1 in Fig. 15, it can be seen from the stress cloud chart of the joint and main part that the simulation phenomenon fits with the test form relatively, the equivalent stress distribution of the specimen is in good agreement with the equivalent stress value measured by the test strain gauge. The test results is emphasized well in the simulation with high reference value.

**Figure 16:**
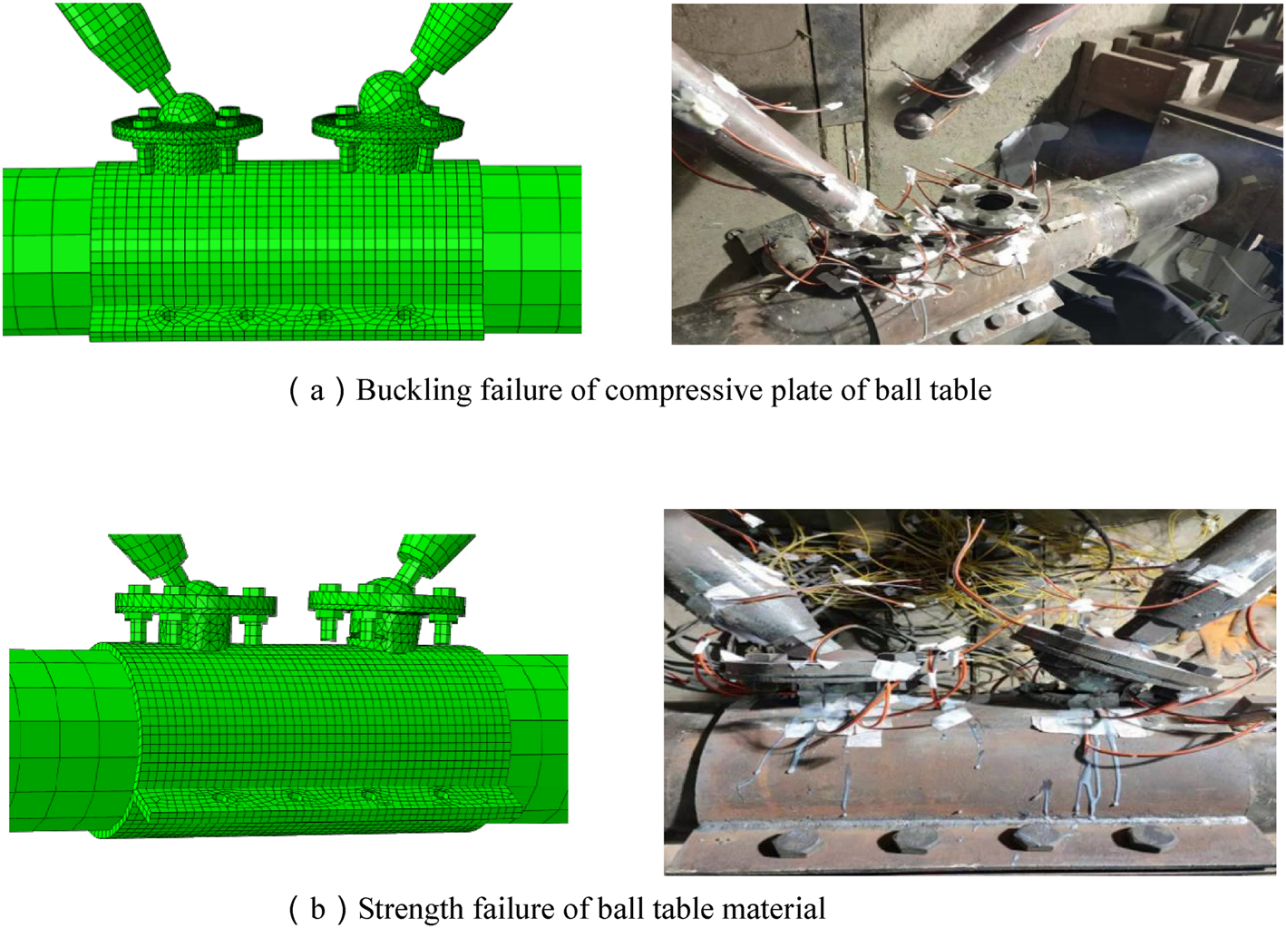
Comparison of failure modes (ABAQUS 2020 https://www.3ds.com/products-services/simulia/).

It can be seen from Fig. 16 that the out-of-plane buckling of the compressive plate of the ball table in the tension zone and the pulling-off of the ball table can be simulated better with Abaqus, which is consistent with the test phenomenon.

### Stress analysis of core concrete column

The radial stiffness of the steel tube is improved greatly from the existence of the concrete in the steel tube for the concrete-filled steel tube structure, thus preventing the local buckling of the steel tube. The stress distribution of the core concrete is not monitored due to the limitation of the test conditions. Moreover, the finite element calculation result for the control group of the specimen JD4 is consistent with that of specimen JD3, therefore, the core concrete columns of the specimens JD1–JD3 are analyzed.

**Figure 17:**
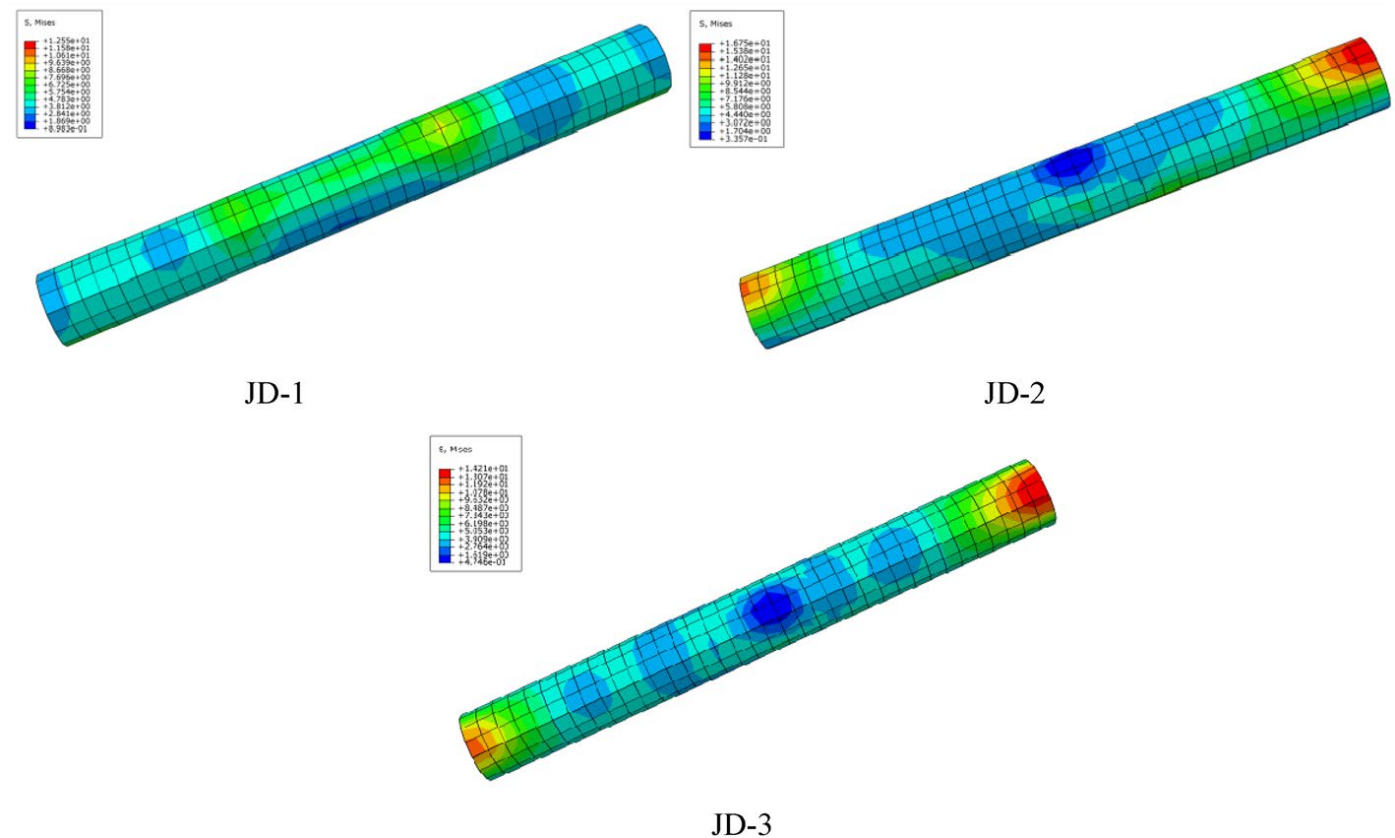
Stress distribution of core concrete column.

**Figure 18:**
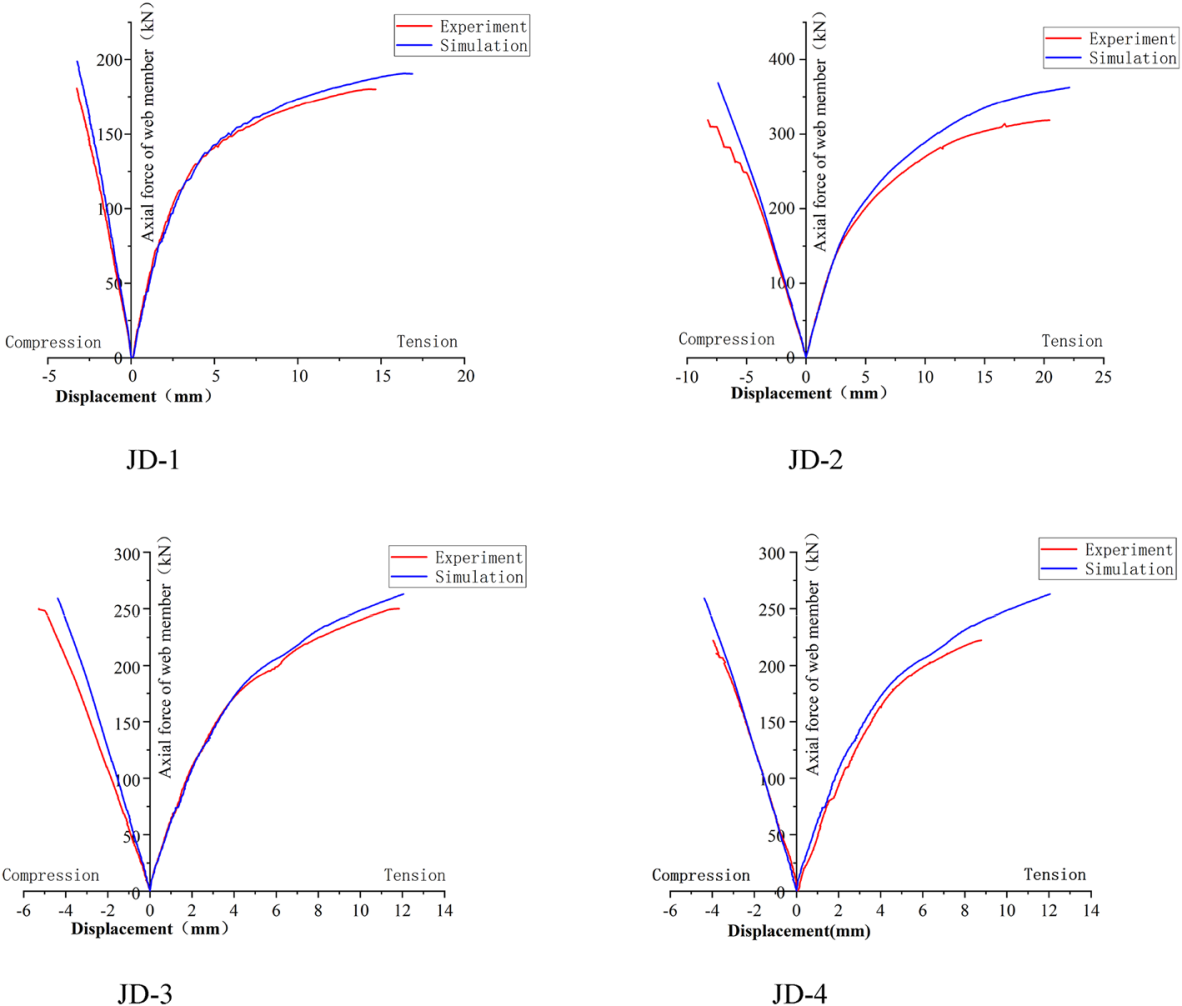
Comparison diagram of load–displacement curve of web member.

The stress distribution of the core concrete column is shown in Fig. 18. It can be seen from Fig. 17 that the maximum stress of the specimens JD1–JD3 is small with the value of 12.5 MPa, 16.7 MPa, 14.2 MPa respectively, and the core concrete column has not been fully utilized.

### Calibration of axial force–displacement curve of web member

Seen from the comparison diagram of load–displacement curve of web member in Fig. 18, we can know that the load–displacement curves of each group of joints are in good agreement, the trend at the early stage is consistent basically, while that at the second half stage is more fuller when compared with the test curve for it is ideal for the modeling and material property in the finite element analysis, and it is not disturbed by the external factor during the simulated loading process.

Seen from Table 7, we can know that the maximum error rate between the test value and the simulated value of the ultimate bearing capacity of the joint is about 10%, thus indicating that the modelling, material property definition, analysis step establishment, interaction setting, load setting and mesh subdivision are reasonable, therefore, the parameter expansion analysis can be carried out.

**Table 7:** Comparison of ultimate bearing capacity.

Specimen number	N_ut_/kN	N_uc_/kN	*e*/%
JD1	180	183.4	1.8
JD2	318	351.4	10.5
JD3	242	252.5	4.3
JD4	229	252.5	10.2

### Parameter expansion analysis

Due to the limited test conditions and specimen number, the expansion analysis of key parameter of the joint is conducted by ABAQUS, 40 finite element analysis models are set up to control the change of two parameters, and the other parameters remain unchanged. The results of parameter expansion analysis are shown in Table 8.

**Table 8:** Parameter expansion.

Thickness of pressure plate/mm	Wall thickness of the ball table/mm	N_uc_/kN	Model number		Thickness of pressure plate/mm	Wall thickness of the ball table/mm	N_uc_/kN
10	4	170.1	1	21	14	8	307.1
10	5	173.4	2	22	14	9	309.7
10	6	176.6	3	23	14	10	314.1
10	7	183	4	24	14	11	319.9
10	8	183.4	5	25	16	4	252.5
10	9	185.4	6	26	16	5	274.7
10	10	186.9	7	27	16	6	313.2
10	11	187.9	8	28	16	7	352.3
12	4	173.9	9	29	16	8	351.4
12	5	192.6	10	30	16	9	365.1
12	6	215.1	11	31	16	10	368.2
12	7	232.4	12	32	16	11	372.3
12	8	240.5	13	33	18	4	255.5
12	9	244.2	14	34	18	5	289.8
12	10	248.9	15	35	18	6	334.1
12	11	252.6	16	36	18	7	368.3
14	4	209.6	17	37	18	8	375.2
14	5	234.4	18	38	18	9	381.1
14	6	264.6	19	39	18	10	385.3
14	7	295.5	20	40	18	11	387.1

### Wall thickness of the ball table

**Figure 19:**
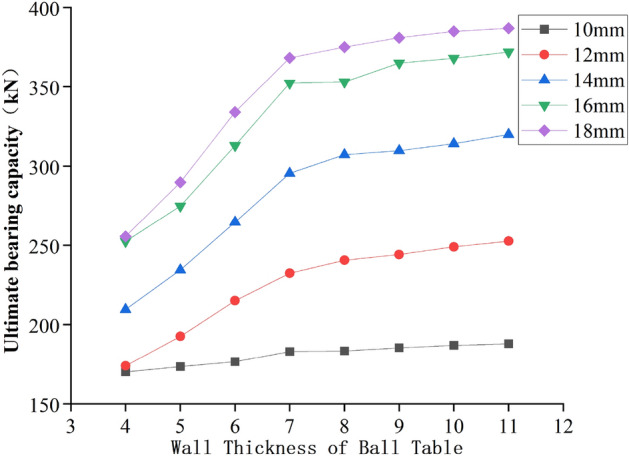
Relationship between ultimate bearing capacity and wall thickness of the ball table.

According to the relationship curve between the ultimate bearing capacity and the wall thickness of the ball table in Fig. 19, we can know that when the thickness of the pressure plate of the ball table is 10 mm, 12 mm, 14 mm, 16 mm and 18 mm respectively, and the wall thickness of the ball table is increased from 4 to 7 mm, the growth range of the ultimate bearing capacity of the joint is 7.6%, 36.6%, 41%, 39.5% and 44.1% respectively, and the ultimate bearing capacity of the joint is increased by 13.1 kN, 39.8 kN, 85.9 kN, 99.8 kN and 112.8 kN respectively; When the wall thickness of the ball table is increased from 7 to 10 mm, the growth range of the ultimate bearing capacity of the joint is 2.1%, 7%, 6.2%, 4.5% and 4.6% respectively, and the ultimate bearing capacity of the joint is increased by 3.9 kN, 16.5 kN, 18.6 kN, 15.9 kN and 17 kN respectively; When the wall thickness of the ball table is increased from 10 to 11 mm, the growth range of the ultimate bearing capacity of the joint is 0.5%, 1.4%, 1.8%, 1.1% and 0.5% respectively, and the ultimate bearing capacity of the joint is increased by 1 kN, 3.7 kN, 5.8 kN, 4.1 kN and 1.8 kN respectively.

To sum up, when the thickness of the pressure plate of the ball table remains unchanged and the bearing capacity is met, and the wall thickness of the ball table is less than 7 mm, the wall of the ball table is thinner, and the failure mode of the joint is the strength failure of the table material. The wall thickness of the ball table is the key parameter affecting the ultimate bearing capacity of the joint, therefore the increase of the wall thickness of the ball table can greatly improve the ultimate bearing capacity of the joint; When the wall thickness of the ball table is greater than 7, the table plate at tension zone is destroyed by the larger loading. At this time, the increase of the wall thickness of the ball table cannot improve the bearing capacity of the table plate at tension zone, and the change of the wall thickness of the ball table has little effect on the ultimate bearing capacity of the joint.

### Thickness of pressure plate of the ball table

**Figure 20:**
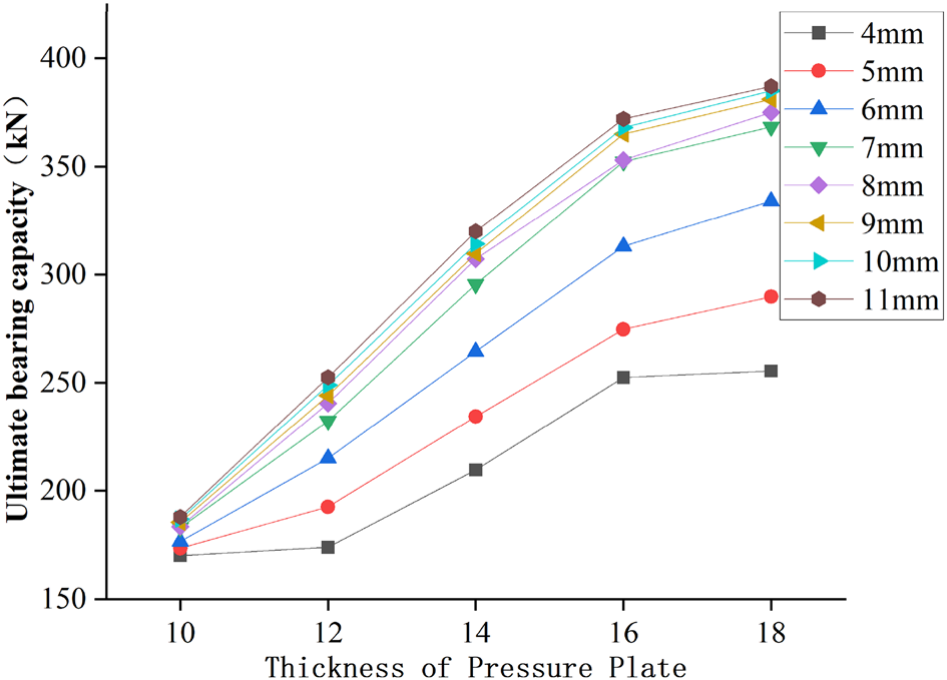
Relationship between ultimate bearing capacity and thickness of pressure plate of the ball table.

It can be seen from Fig. 20 that when the wall thickness of the ball table is 4 mm, 6 mm, 8 mm and 10 mm respectively, and the thickness of the pressure plate of the ball table is increased from 10 to 12 mm, the growth range of the ultimate bearing capacity of the joint is 2.2%, 21.8%, 31.1%, 33.1% respectively, and the ultimate bearing capacity of the joint is increased by 3.8 kN, 38.5 kN, 57.1 kN and 62 kN respectively; When the thickness of the pressure plate of the ball table is increased from 12 to 14 mm, the growth range of the ultimate bearing capacity of the joint is 20.5%, 23%, 27.6%, 26.1% respectively, and the ultimate bearing capacity of the joint is increased by 35.7 kN, 49.5 kN, 66.6 kN and 65.2 kN respectively; When the thickness of the pressure plate of the ball table is increased from 14 to 16 mm, the growth range of the ultimate bearing capacity of the joint is 20.4%, 18.4%, 14.4%, 17.2% respectively, and the ultimate bearing capacity of the joint is increased by 42.9 kN, 48.6 kN, 44.3 kN and 54.1 kN respectively; When the thickness of the pressure plate of the ball table is increased from 16 to 18 mm, the growth range of the ultimate bearing capacity of the joint is 1.1%, 6.6%, 6.7%, 4.6% respectively, and the ultimate bearing capacity of the joint is increased by 3kN, 20.9kN, 23.8 kN and 17.1 kN respectively.

To sum up, when the wall thickness of the ball table remains unchanged and the table wall meets the bearing capacity, and the thickness of the pressure plate of the ball table is less than 16 mm, the pressure plate of the ball table is thinner, and the failure mode of the joint is the buckling failure of the pressure plate of the ball table at tension zone. At this time, the thickness of the pressure plate of the ball table is the key parameter affecting the ultimate bearing capacity of the joint, therefore, the increase of the thickness of the pressure plate of the ball table can greatly improve the ultimate bearing capacity of the joint; When the thickness of the pressure plate of the ball table is greater than 16 mm, the table plate at tension zone is destroyed by the greater loading. At this time, the increase of the thickness of the pressure plate of the ball table cannot improve the bearing capacity of table plate at tension zone, and the change of the thickness of pressure plate of the ball table has little effect on the ultimate bearing capacity of the joint.

The two parameters of the thickness of the ball table plate and the thickness of the ball table wall are researched in the paper. In order to explore the influence of the inclusion, the web member and the thickness of tower column on the joint, the thickness of the ball table plate is 16 mm and the wall thickness of the ball table is 8 mm. 7 finite element analysis models are set up by ABAQUS, and the analysis results are shown in Table 9.

**Table 9:** Parameter expansion.

Model number	Thickness of pressure plate/mm	Wall thickness of the ball table/mm	Cross section of tower	Cross section of web member	Cross section of wrapped object	Nuc/kN
1	16	8	φ219 × 6	φ89 × 5	φ235 × 8	351.4
2			φ219 × 7	φ89 × 5	φ236 × 8	352.8
3			φ219 × 8	φ89 × 5	φ237 × 8	354.3
4			φ219 × 6	φ89 × 6	φ235 × 8	353.6
5			φ219 × 6	φ89 × 7	φ235 × 8	355.1
6			φ219 × 6	φ89 × 5	φ235 × 9	353.2
7			φ219 × 6	φ89 × 5	φ235 × 10	354.6

It can be seen from Table 9 that when the thickness of the pressure plate is 16 mm and the thickness of the wall is 8 mm, the wall thickness of the tower column increases from 5 to 7 mm, and the ultimate bearing capacity of the joint increases by 0.82%. When the wall thickness of web member increases from 4 to 6 mm, the ultimate bearing capacity increases by 1.1%. When the wall thickness of the inclusion increases from 7 to 9 mm, the ultimate bearing capacity of the joint increases by 0.91%.

In summary, when the thickness of the ball table plate and the wall thickness of the ball table are unchanged the tower column, inclusions and web members meet the bearing capacity, the ultimate bearing capacity of the joint is also increased to a certain extent, but the effect is small along with the increase of the wall thickness of the tower column, inclusions and web members. The reason is that the tower column mainly plays a local pressure-bearing role in the whole process, and the web members and inclusions do not exert their full working performance when compared with the platform area. The increase of stiffness does not match the overall stiffness of the joint, the platform plate and the platform wall are destroyed firstly. It also further illustrates that the thickness of the table pressure plate and the table wall are two key parameters affecting the node.

## Conclusions

1. The failure of the joints can be divided into two modes, namely, the buckling failure of the pressure plate and the strength failure of the table material. The buckling failure of the pressure plate is a kind of plastic failure, and the strength failure of the table material is a type of brittle failure.
2. The wall thickness of the ball table is the key parameter affecting the failure mode of the joint. When the thickness of the pressure plate of the ball table remains unchanged, increasing the wall thickness of the ball table is an effective way to improve the failure mode of the joint. The pressure plate of the ball table is a key parameter affecting the ultimate bearing capacity of joints. When the wall thickness of the ball table is 8 mm and the thickness of the pressure plate of the ball table is increased from 10 to 16 mm, the ultimate bearing capacity of the specimen is increased by 77.2%.
3. The increase of the thickness of the bearing plate can effectively improve the ultimate bearing capacity of the joint, and the increase of the thickness of the wall can effectively improve the overall deformation capacity of the joint and give full play to the ductility of the joint.
4. Compared with the abutment wall and the abutment pressure plate, the tower column, the web member and the inclusion have less influence on the joint. It is suggested to change the section parameters appropriately in practical engineering under the premise of meeting the bearing capacity.
5. The stresses at tension and compression zone of the joint are not coordinated, and the ball table in the compression zone is only subjected to a small part of the pressure, while most of the load in the tension zone is borne by the ball table itself. The pressure plate of the ball table in the compression zone is relatively weak on the side squeezed by the bolt ball, and it is recommended to design the reinforcement device in the actual project.
6. When the wall thickness of the ball table is greater than 7 mm, the thickness of the pressure plate of the ball table is greater than 16 mm, the growth rate of the ultimate bearing capacity of the joint is significantly decreased. In order to ensure the safety and economy of the joint, it is recommended to use the wall thickness of 7 mm and the pressure plate thickness of 16 mm in the actual design of the project.

## Data Availability

The datasets generated and/or analysed during the current study are not publicly available due fund confidentiality but are available from the corresponding author on reasonable request.
